# Bibliography

**Published:** 1903-11

**Authors:** 


### Bibliography.

A Dictionary of Medical Science, containing a Full Explanation of the Various Subjects and Terms of Anatomy, Physiology, Medical Chemistry, Pharmacy, etc. By Robley Dunglison, M.D., LL.D., late Professor of Institutes of Medicine and Medical Jurisprudence in the Jefferson Medical College of Philadelphia, etc. Twenty-third edition. Thoroughly revised, with the pronunciation, accentuation, and derivation of terms. By Thomas L. Stedman, A.M., M.D., Fellow of the New York Academy of Medicine. Lea Brothers & Co., Philadelphia and New York, 1903.

The presentation of a twenty-third edition of Dunglison's Dictionary carries the mind back so far in the history of medicine that probably very few are sufficiently advanced in years to remember the earlier editions of this great work. The reviser says of it, "A book which has held its own through twenty-two editions for nearly three-quarters of a century needs no apology. . . . It has become an institution, and the only office of the present editor is to tell what liberties he has taken with the work of the author, and why."

The reviewer has before him the third edition of this dictionary published by Lea & Blanchard in 1842. In the preface of the second edition, republished in the third, the author, Dr. Dunglison, says, "The author's object has not been to make the work a mere lexicon or dictionary of terms, but to afford under each a condensed view of its medical relations, and thus to render the work an epitome of the existing condition of medical science." This original idea of the author has been faithfully followed in each successive edition until the present, and the student of the history of medicine can readily follow the steps of progress made, for nothing so marks this as the new words added to indicate new phases of the science as they have appeared from decade to decade.

Turning to the third edition of 1842, the word bacillus has no place, or bacteriology developed out of this. Probably no single word more prominently represents the progress made in Medicine than this. In the twenty-third, the present edition, some fifteen double-column pages of text are devoted to this subject, and four pages of illustrations. That which is true of this word is measurably so of other terms. The reviser states that it has been found necessary to add new terms "varying in number from several hundred to several thousand."

Among the words that have required more extended definition is that of Anæsthesia. In the 1842 edition the definition of this word is confined to "Privation of sensation, and especially that of touch. . . . It may be general or partial." This edition was published two years before Wells discovered the anæsthetic properties of nitrous oxide and gave a practical illustration of it, and four years prior to Morton and Jackson's experiments with ether. The definition is practically that given by Gardiner in his 1847 dictionary, and also the older dictionaries; as, for instance, Parr's Medical Dictionary, 1819, defines the word in the same way as those quoted. This definition dates back to Cullen, and was not practically changed until anæsthesia meant reduction of pain by inhalation. It is a curious fact that a very general impression prevails that this word was introduced by Dr. Oliver Wendell Holmes at the time ether was introduced. Holmes does not claim it, for he alludes in his letter, 1846, to Cullen and Linnæus in this connection, but he evidently had doubts of the propriety of applying this word in the modern sense, for he recommends Morton to consult with certain "accomplished scholars, such as President Everett or Dr. Bigelow, before fixing upon the term which will be repeated by the tongues of every civilized race of mankind."

The reviser has been careful to include words used in dentistry, and an examination of these gives, upon the whole, satisfactory definitions, although, in general, these might have been extended to advantage in a somewhat similar manner to those regarded of importance in medicine. In illustration, under "Dental" many of the instruments used in dentistry are described so briefly that one not familiar with the subject would have some difficulty in determining their use; for instance, the word "Dental model." It is defined as a "plaster cast which is to be accurately reproduced in other material." This will strike the average dentist as absurd. For one not conversant with the use of a model it may be necessary to add that it is simply a form reproduced from an impression of the mouth, which in its turn is used to form a plate for the mouth upon which to attach teeth; in other words, to produce an artificial denture, or for other mechanical appliances.

The reviser has introduced the word "morsal," meaning bite. This has not been generally adopted in dentistry, and probably will not take the place of occlusal, now generally used to convey the same idea.

Those who have regarded Dunglison's Dictionary as a medical authority, and have reverenced it not only from long association, but for its intrinsic value, will have this long-time regard strengthened by reference to this edition. The reviser has performed his part of the work very satisfactorily, and although there are now many dictionaries of words used in medicine, and all of them of very great value, this will hold a place second to no other in the library of the practitioner, or in the daily work of the student in general medicine or of any of its branches.

The publishers have made this work more valuable by carefully prepared illustrations and have also made reference easy by marginal lettering.

